# Supplementary material for: Hybrid Models and Biological Model Reduction with PyDSTool
Source: PLoS Comput Biol. 2012 Aug 9;8(8):e1002628. doi: 10.1371/journal.pcbi.1002628 (PMC3415397; doi:10.1371/journal.pcbi.1002628)
Supplement: Text S4 — Complete source code for the PyDSTool package (version 0.88.120504). Includes API documentation and help files linking to web pages. This file is identical to the current public release on Sourceforge.net. (ZIP) [file pcbi.1002628.s004.zip › PyDSTool/html/PyDSTool.Trajectory'.HybridTrajectory-class.html]

xml version="1.0" encoding="ascii"?


PyDSTool.Trajectory'.HybridTrajectory


| Home | Trees | Indices | Help | PyDSTool |
| --- | --- | --- | --- | --- |

Package PyDSTool ::
Module Trajectory' ::
Class HybridTrajectory

[hide private]

[frames] | no frames]

# Class HybridTrajectory

source code

```
object --+    
         |    
Trajectory --+
             |
            HybridTrajectory
```

---

Hybrid, parameterized, trajectory class. Mimics API of a non-hybrid
Trajectory.

vals must be a sequence of trajectory segments. coords is optional
(restricts variables to store).


|  |  |
| --- | --- |
| Instance Methods | [hide private] |

|  |  |
| --- | --- |
| \_\_call\_\_(self, t, coords=None, checklevel=0, asGlobalTime=False, asmap=False)  Evaluate a parameterized hybrid trajectory at given independent variable value(s), interpreted as global times if optional asGlobalTime==True (default is False). | source code |

|  |  |
| --- | --- |
| \_\_init\_\_(self, name, vals, coordnames=None, modelNames=None, timeInterval=None, modelEventStructs=None, eventTimes=None, events=None, FScompatibleNames=None, FScompatibleNamesInv=None, abseps=None, globalt0=0, checklevel=0, norm=2, timePartitions=None)  x.\_\_init\_\_(...) initializes x; see x.\_\_class\_\_.\_\_doc\_\_ for signature | source code |

|  |  |
| --- | --- |
| info(self) | source code |

|  |  |
| --- | --- |
| sample(self, coords=None, dt=None, tlo=None, thi=None, doEvents=True, precise=False, asGlobalTime=True)  Uniformly sample the trajectory over range indicated, including any event points specified by optional 'doEvents' argument (default True). | source code |

|  |  |
| --- | --- |
| showRegimes(self) | source code |

|  |  |
| --- | --- |
| underlyingMesh(self, coords=None, FScompat=True)  Return a dictionary of the underlying independent variables` meshes, where they exist. | source code |

**Inherited from `Trajectory`**:
`__copy__`,
`__deepcopy__`,
`__del__`,
`__getstate__`,
`__repr__`,
`__setstate__`,
`__str__`,
`delete_variables`,
`getEventTimes`,
`getEvents`,
`mapNames`,
`truncate_to_idx`,
`truncate_to_indepvar`

**Inherited from `Trajectory`** (private):
`_createEventTimes`,
`_infostr`

**Inherited from `object`**:
`__delattr__`,
`__getattribute__`,
`__hash__`,
`__new__`,
`__reduce__`,
`__reduce_ex__`,
`__setattr__`


|  |  |
| --- | --- |
| Properties | [hide private] |

**Inherited from `object`**:
`__class__`


|  |  |
| --- | --- |
| Method Details | [hide private] |

|  |  |
| --- | --- |
| \_\_call\_\_(self, t, coords=None, checklevel=0, asGlobalTime=False, asmap=False)  *(Call operator)* | source code |

Evaluate a parameterized hybrid trajectory at given independent
variable value(s), interpreted as global times if optional
asGlobalTime==True (default is False).

asmap option allows the trajectory to be called as a map, with t =
integer values to return the values at successive hybrid event times.
Result includes actual time values (in global time if
asGlobalTime==True).

Overrides: Trajectory.\_\_call\_\_

|  |  |
| --- | --- |
| \_\_init\_\_(self, name, vals, coordnames=None, modelNames=None, timeInterval=None, modelEventStructs=None, eventTimes=None, events=None, FScompatibleNames=None, FScompatibleNamesInv=None, abseps=None, globalt0=0, checklevel=0, norm=2, timePartitions=None)  *(Constructor)* | source code |

x.\_\_init\_\_(...) initializes x; see x.\_\_class\_\_.\_\_doc\_\_ for
signature

Overrides: object.\_\_init\_\_: *(inherited documentation)*

|  |  |
| --- | --- |
| info(self) | source code |

Overrides: Trajectory.info

|  |  |
| --- | --- |
| sample(self, coords=None, dt=None, tlo=None, thi=None, doEvents=True, precise=False, asGlobalTime=True) | source code |

```
Uniformly sample the trajectory over range indicated,
  including any event points specified by optional 'doEvents'
  argument (default True).
Returns a pointset.

precise=False attempts to use the underlying mesh of the trajectory
  to return a Pointset more quickly. Currently, this can only be used
  for trajectories that have a single segment.

If dt is not given, the underlying time mesh is used, if available.

asGlobalTime is ignored for this class (time is always global for
  this class).
```

Overrides: Trajectory.sample

|  |  |
| --- | --- |
| underlyingMesh(self, coords=None, FScompat=True) | source code |

Return a dictionary of the underlying independent variables` meshes,
where they exist. (Dictionary contains references to the meshes, not
copies of the meshes.) Always returns data in local time frame.

FScompat option selects whether to use FuncSpec compatible naming for
variables (mainly for internal use).

Overrides: Trajectory.underlyingMesh

  


| Home | Trees | Indices | Help | PyDSTool |
| --- | --- | --- | --- | --- |

|  |  |
| --- | --- |
| Generated by Epydoc 3.0.1 on Fri May 4 15:24:10 2012 | http://epydoc.sourceforge.net |
